# An abelisaurid (Dinosauria: Theropoda) ilium from the Upper Cretaceous (Cenomanian) of the Kem Kem beds, Morocco

**DOI:** 10.1371/journal.pone.0214055

**Published:** 2019-04-02

**Authors:** Slimane Zitouni, Christian Laurent, Gareth Dyke, Nour-Eddine Jalil

**Affiliations:** 1 Département de Géologie, Université Cadi Ayyad, Marrakech, Morocco; 2 Muséum d’Histoire Naturelle de Marrakech, (MHNM), Marrakech, Morocco; 3 Ocean and Earth Science, National Oceanography Centre, Southampton, United Kingdom; 4 Department of Geology, Babes-Bolyai University, Cluj-Napoca, Romania; 5 Department for Evolutionary Zoology, University of Debrecen, Debrecen, Hungary; 6 Muséum national d'Histoire naturelle, Sorbonne Université, Paris, France; State Museum of Natural History, GERMANY

## Abstract

Abelisaurid theropods first appear in the fossil record in the early Jurassic and survived at least until the end of the Mesozoic. They were known to have dominated South America, India and Madagascar but were not so abundant in North America or Asia. Much less is known about their presence in Africa, although there has been several recent discoveries of abelisaurid material in Morocco. Here we add a partially preserved ilium to a growing body of evidence that suggests abelisaurs might also have dominated Africa.

## Introduction

Abelisaurid theropods first appear in the fossil record in the early Jurassic and survived until at least the end of the Mesozoic [1–3]. These dinosaurs were dominant in South America, India, and Madagascar but are thought to have been less abundant in North America and Asia [3].

These dinosaurs are much less well-known in Africa, although several recent discoveries of abelisaurid fossils have been made in North Africa [2,4–11]. In this work, we add a partially preserved ilium to a growing body of evidence that suggests that abelisaurs were also present, and dominant in Africa. This material was recovered from the surface at the Aferdou site, near the Gara Sbaâ locality, Morocco. This locality is situated within the Kem Kem beds, which outcrop in the lower Cenomanian of Eastern Morocco.

Most fossil material recovered from this region is poorly preserved as fossils are normally recovered from the surface and may have been reworked. As a result, the bones are sometimes eroded, fragmented or incomplete. Moreover, in the last few decades the fossil trade has played an increasingly important role in local economy, and this is especially true for fossils from the Upper Cretaceous. Many fossils are damaged because of poor collection practices and in most cases, there is no precise information on location, sedimentology or stratigraphy. Specimens or fragments that are thought to be of no commercial value are often neglected and become unrecoverable [12]. However, this is not always the case and some more careful collectors recover better preserved fossils and provide relevant provenance information.

Generally, theropod remains are more abundant than sauropod remains in the Kem Kem beds and no ornithischian material has been recovered here at all. Although, some ornithischian footprints are reported by Sereno *et al*. [13] and Belvedere *et al*. [14]and were recently described by Ibrahim *et al*. [5].

### Geology and stratigraphy

**Fig 1:**
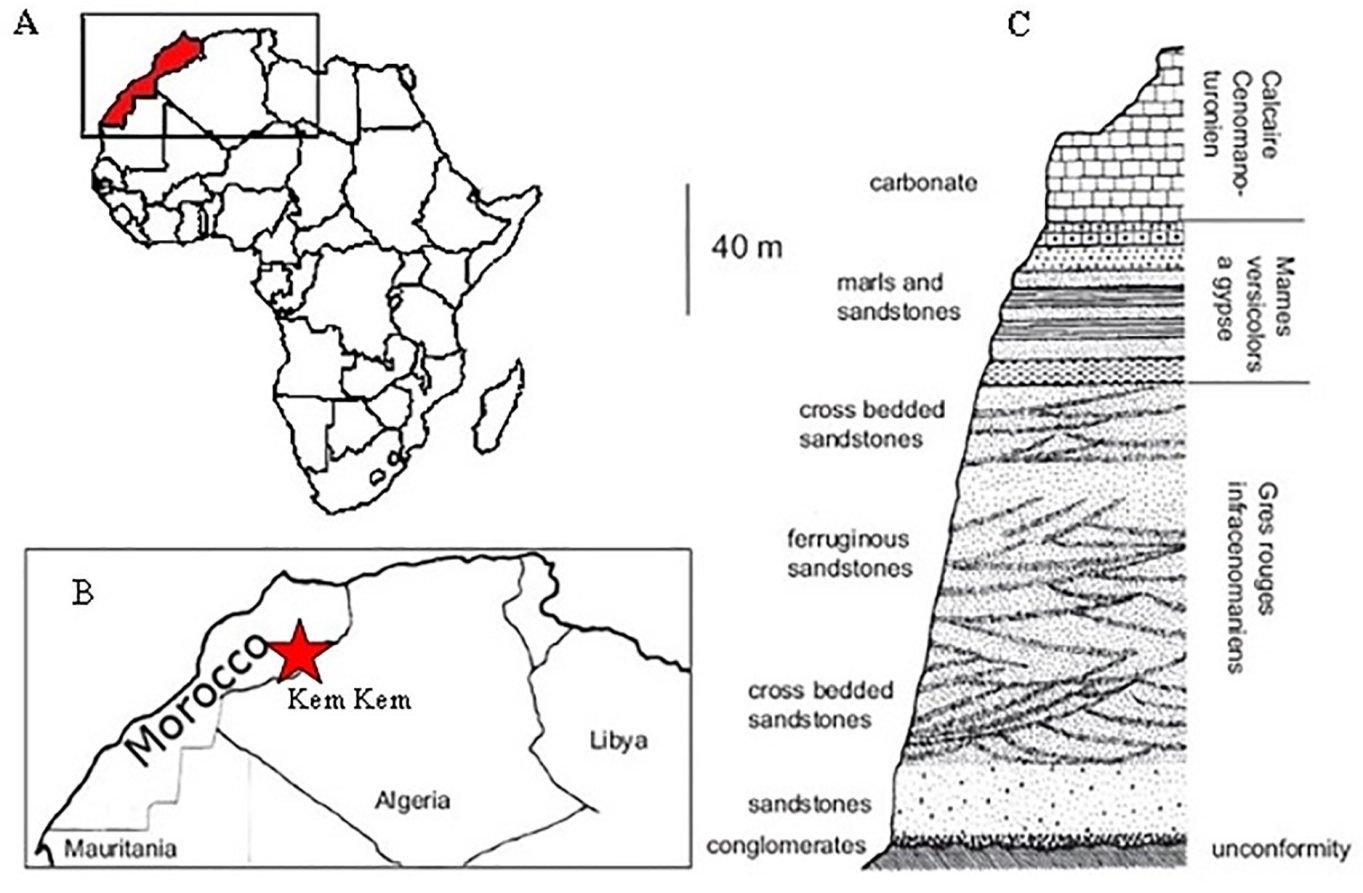
Location and stratigraphy of the Kem Kem beds. **A** and **B** Location of the Kem Kem beds in Morocco; **C** Kem Kem stratigraphy (modified from Russell [4]).

The Kem Kem region in Eastern Morocco comprises an extensive series of red terrestrial sandstone deposits that are Upper Cretaceous in age. These deposits are known worldwide as the Kem Kem beds and are located within the Tafilalt region of eastern Morocco (Fig 1A and 1B). This sequence, initially considered as Cretaceous Infracenomanian (Albian) [4,15–17]; is currently dated Lower Cenomanian on the basis of an assembly of selacean teeth [13,18] and by comparison with other vertebrate fossils in North Africa [19]. Outcrops of the Kem Kem beds lie non-conformably on the Palaeozoic basement [15]; Dubar [20] subdivided the deposits of these beds into three successive formations (a trilogy) (Fig 1C) which was later confirmed by Ettachfini & Andreu [21]. This trilogy spans from the base of the Ifezouane Formation upwards, and consists mainly of red, criss-crossed sandstones that suggest a continental river deposit. The overlying unit, the Aoufous Formation, consists mostly of clay-sandstone containing green marl gypsum.

These lowermost formations of this Lower Cenomanian trilogy correspond to the “Intercalar Continental” of Kilian [22] and are between 100 m and 150 m thick [23]. The last formation of the trilogy is the carbonaceous Akrabou Formation which overlies conformably the Aoufous Formation. It corresponds to a very large slab of Cenomano-Turonian limestone (Fig 1C). The Lower Cenomanian red sandstone formations are exposed for approximately 250 km following a curvature east and south of Erfoud, along the border with Algeria.

## Material and methods

During fieldwork conducted in April 2007 by GD and NEJ, a number of incomplete and fragmented skeletal remains were discovered and collected or bought. The ilium described in this work is from the commercial network and was bought directly from its collector. It was discovered completely clear at the surface within the Aferdou region, near the locality of Gara Sbaâ (30°32′22″ N; 04°50′23″ W). The ilium consists of several pieces that have been prepared, assembled and deposited in the Natural History Museum of Marrakech (MHNM), at Cadi Ayyad University, Morocco, under collection number MHNM KK04.

## Results and discussion

### Description

The ilium is highly fractured and fragile. It was discovered completely clear of matrix and consists of several pieces. Once these pieces had been cleaned and re-assembled it became clear that the iliac blade is incomplete and the anterior process is damaged. The underside of the pubic peduncle is also damaged. Such damage is undoubtedly due to awkwardness when collecting, as shown by the fresh surface of the fractures at the dorsal border of the iliac plate.

**Fig 2:**
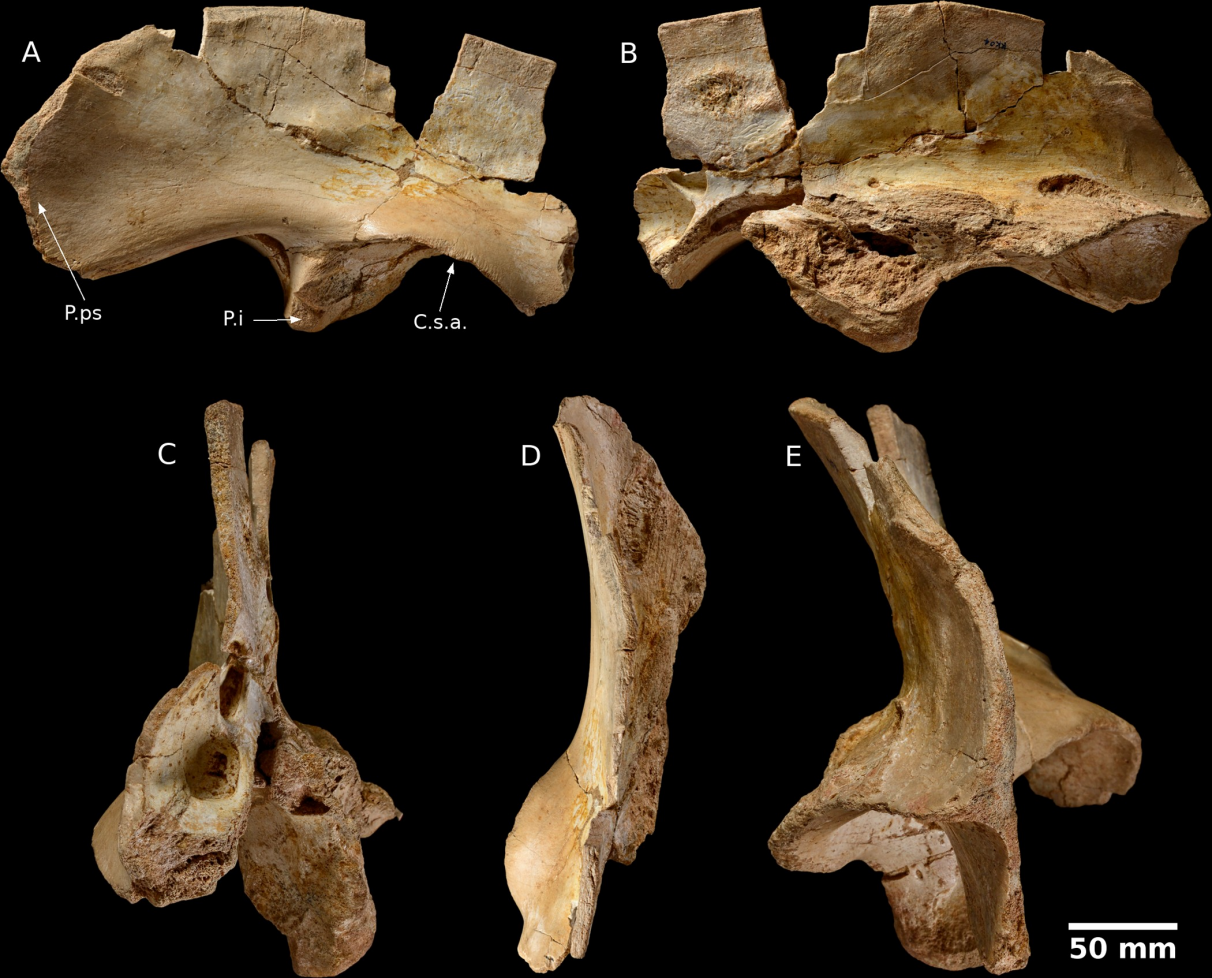
Abelisaur (indeterminate), right ilium (MHNM KK 04). **A** lateral view; **B** medial view; **C** Anterior; **D** dorsal and **E** Posterior view. Abbreviations: Csa, supra-acetabular abutment; Pi, ischial peduncle; P.ps posterior process. Scale bar 10 cm.

**Lateral view** (Fig 2A)—Almost all of the anterior process is absent, except for a fracture in a small piece which extends beyond the pubic peduncle. The posterior process is very long, sub-rounded and extends behind the ischial peduncle. The lateral face of this process has a relatively smooth, flat surface and its transverse thickness is relatively thin.

The supra-acetabular buttress is incomplete and the process which connects with the pubic peduncle is missing. It is well developed laterally and extends posteriorly to the ischial peduncle. The surface of the acetabulum is oriented ventrally. The blade above the acetabulum is fractured, but its general appearance is easy to identify. It shows a concavity on the lateral surface on the ventral side and the sub-plane side of the dorsal edge with a slight bulge at the centre. This blade is formed of a thinner bone transversely and extends posteriorly to the posterior process.

The pubic peduncle is badly damaged and only the ventral part is preserved. Otherwise, the ischial peduncle is relatively well preserved and shows less damage than other parts of this fossil but it is underdeveloped ventrally as well as anteroposteriorly. The total length of the ischial peduncle is smaller than its transverse width. The edge of the ilium between the ischial peduncle and posterior process is especially curved and opens ventrally.

**Medial view** (Fig 2B)—The surface is quite irregular at the ischial peduncle, it is generally smooth and has a slight anteroposterior convexity at the posterior process and the iliac blade. The surface has an elongate wrinkle that extends from the centre of the ventral side of the iliac blade to the posterior end of the posterior process. The dorsal surface is flatter with no observable convexity. This relief may correspond to the contact surface between the ilium and sacral vertebrae.

**Anterior, Dorsal and Posterior views** (Fig 2C, 2D and 2E)—The dorsal edge of the ilium is very fine and suggests a fragile bone. The general profile of the bone shows a curvature of the lateral side due to the low concavity of the iliac blade.

### Discussion

The post-acetabulum part of the iliac blade is sub-rounded. It has a semi-circular shape, and is not truncated as is the case with *Allosaurus*, *Sinraptor*, *Suchomimus*, *Baryonyx*, nor tapered like those of *Torvosaurus* or *Eustreptospondylus*. The iliac blade is much like that of other theropods, but it is relatively long and resembles *Ichthyovenator laosensis* [24].

Almost two thirds of characters from the ilium which are normally used in the most important phylogenetic classifications are based on the pubic peduncle, the anterior process and the anterior side of the iliac blade. Since this part is poorly preserved in MHNM KK04, it is difficult to determine its affinity with precision. However, the ilium does preserve some derived characters that diagnose some theropod clades. The *brevis fossa* is located on the ventral edge of the postacetabular blade of the ilium. It is relatively deep in all theropods [25] except for *Herrerasaurus* [26] and *Eoraptor* [27] and so diagnoses the clade Neotherapoda Bakker, 1986 [28]. The *brevis fossa* is also broadened posteriorly [2,7,29–32]. This broadening is a derived trait which has been observed in the following taxa: *Carnotaurus*, *Ceratosaurus*, *Ligabueino*, *Majungasaurus*, and *Masiakasaurus*. This synapomorphy usually determines the clade Ceratosauria. Another derived character is the shape of the dorsal margin of the ilium (without the posterior and anterior process) [2,7,29,30]. This character is a shared synapomorphy of the following taxa: *Carnotaurus*, *Deltadromeus*, *Ligabueino*, *Majungasaurus*, *Masiakasaurus* and *Skorpiovenator* and determines the clade Abelisauria.

Although spinosaurid fossils are common in the Kem Kem [5,11,33–37] we do not consider this assignment likely for this ilium. There has been no detailed description of a spinosaurid ilium but there are notable differences between MHNM KK04 and the spinosaurid ilium presented by Ibrahim *et al*. [5] (their Fig 2F). MHNM KK04 has a longer posterior process and the ischial peduncle is much less developed both ventrally and anteroposteriorly [34].

On the basis of the synapomorphies considered above and the differences between MHNM KK04 and *Spinosaurus*, we consider MHNM KK04 an indeterminate Abelisaur. Unfortunately, the state of preservation in the fossils of the Kem Kem beds does not usually allow advanced comparisons with other, better known theropod groups.
